# Identification of a centrosome-related prognostic signature for breast cancer

**DOI:** 10.3389/fonc.2023.1138049

**Published:** 2023-03-22

**Authors:** Zhou Fang, Zhi-Jie Gao, Xin Yu, Sheng-Rong Sun, Feng Yao

**Affiliations:** Department of Breast and Thyroid Surgery, Renmin Hospital of Wuhan University, Wuhan, Hubei, China

**Keywords:** breast cancer, centrosome, CRSS, prognostic model, immune, machine algorithm

## Abstract

**Background:**

As the major microtubule organizing center in animal cells, the centrosome is implicated with human breast tumor in multiple ways, such as promotion of tumor cell immune evasion. Here, we aimed to detect the expression of centrosome-related genes (CRGs) in normal and malignant breast tissues, and construct a novel centrosome-related prognostic model to discover new biomarkers and screen drugs for breast cancer.

**Methods:**

We collected CRGs from the public databases and literature. The differentially expressed CRGs between normal and malignant breast tissues were identified by the DESeq2. Univariate Cox and LASSO regression analyses were conducted to screen candidate prognostic CRGs and develop a centrosome-related signature (CRS) to score breast cancer patients. We further manipulated and visualized data from TCGA, GEO, IMvigor210, TCIA and TIMER to explore the correlation between CRS and patient outcomes, clinical manifestations, mutational landscapes, tumor immune microenvironments, and responses to diverse therapies. Single cell analyses were performed to investigate the difference of immune cell landscape between high- and low-risk group patients. In addition, we constructed a nomogram to guide clinicians in precise treatment.

**Results:**

A total of 726 CRGs were collected from the public databases and literature. *PSME2*, *MAPK10*, *EIF4EBP1* were screened as the prognostic genes in breast cancer. Next, we constructed a centrosome-related prognostic signature and validated its efficacy based on the genes for predicting the survival of breast cancer patients. The high-risk group patients had poor prognoses, the area under the ROC curve for 1-, 3-, and 5-year overall survival (OS) was 0.77, 0.67, and 0.65, respectively. The predictive capacity of CRS was validated by other datasets from GEO dataset. In addition, high-risk group patients exhibited elevated level of mutational landscapes and decreased level of immune infiltration, especially T and B lymphocytes. In terms of treatment responses, patients in the high-risk group were found to be resistant to immunotherapy but sensitive to chemotherapy. Moreover, we screened a series of candidate anticancer drugs with high sensitivity in the high-risk group.

**Conclusion:**

Our work exploited a centrosome-related prognostic signature and developed a predictive nomogram capable of accurately predicting breast cancer OS. The above discoveries provide deeper insights into the vital roles of the centrosome and contribute to the development of personalized treatment for breast cancer.

## Introduction

Currently, breast cancer remains the major threat to women’s health worldwide, and according to statistics, it has become the most prevalent malignancy worldwide (1). Breast cancer is considered as one of highly heterogeneous malignancies with diverse pathologic features and molecular subtypes. Although more and more new targets, drugs, superior medical diagnostic and imaging techniques for breast cancer have emerged (2, 3), the prognosis for breast cancer patients still remain unsatisfying (4, 5). Accumulating researches have indicated that advanced or high-risk breast cancer patients suffer from poor prognoses and treatment outcome (6). The current validated markers and diagnostic tools fail to accurately predict the prognosis and treatment response of breast cancer (7). Therefore, it is imperative to develop novel and effective markers and predictive models.

Centrosomes, the major microtubule nucleating organelles in animal cells, play a key role in mitotic spindle orientation and genome stabilization (8). Cancer cells driven by cytokinesis failure exhibit centrosome amplification (CA), which is an important hallmark of cancer (9). In addition to its well-known role, several recent studies have shown that centrosomes are intimately linked to the genome and immunity. For example, early amplification of centrosomes is dependent on wild-type expression of the tumor suppressor *p53* and hotspot mutations (10). Researches have also shown that the absence of the *BRCA1* oncogene causes centrosome dysregulation that promotes tissue-specific carcinogenesis (11). In addition, centrosome defects have been shown to promote immune escape of tumor cells, which would lead to tumor deterioration (12). Several studies have shown that CA promotes the development of breast cancer (13–16). In breast cancer, CA is seen as a driver of chromosomal instability and breast carcinogenesis. The lowest CA was found in normal breast tissues, and a significantly increased CA was shown in precancerous tissues, while the ductal carcinoma *in situ* and infiltrative tumors exhibited the highest CA (17). For a long time, researchers have intensively studied the role of centrosomes in cancer development and progression. However, to date, there are no relevant studies on centrosome-related prognostic model of breast cancer. Therefore, studying centrosome-related gene markers in breast cancer may provide valuable therapeutic guidance in clinical practice.

In our study, we collected 727 centrosome-related genes (CRGs) from the public databases and literature, and identified the 155 differentially expressed CRGs between normal and cancerous breast samples in TCGA dataset. We then used univariate Cox regression and Least Absolute Shrinkage Selection Operator (LASSO) regression analyses to screen three prognostic CRGs in breast cancer patients, including *PSME2*, *MAPK10*, and *EIF4EBP1*. Based on these three genes, we constructed a centrosome-related prognostic model and centrosome-related signature score (CRSS) for breast cancer patients. According to the CRSS, we classified breast cancer patients into high-risk and low-risk groups. We confirmed that the high-risk group showed a poorer prognosis and a higher frequency of genomic mutations, as well as a lower level of immune infiltration. Regarding to the cellular component at single-cell resolution, the immune cell infiltration differed significantly between high- and low-risk groups. Patients in the high-risk group exhibited decreased composition of immune cell, especially T and B lymphocytes. Additionally, we revealed that patients in the high-risk group were resistant to immunotherapy but sensitive to chemotherapy. A nomogram was conducted to predict the outcome of breast cancer patients. Taken together, this prognostic model provides us with a novel and powerful reference for diagnosis and treatment of breast cancer.

## Materials and methods

### Acquisition and preprocessing of breast cancer datasets

#### Training set

We selected 1128 patients from the Cancer Genome Atlas (TCGA) database and downloaded their mRNA expression matrix and clinical information from UCSC Xena (https://xena.ucsc.edu/). The expression matrix included the fragments per kilobase of exon model per million mapped fragments (FPKM) and the count value. After removing 9 patients with duplicate or incomplete follow-up information, 1020 TCGA breast cancer patients and 99 normal patients were included in our training cohort.

We downloaded somatic mutation data from Genomic Data Commons (GDC, https://portal.gdc.cancer.gov/). Somatic mutation data sorted in the form of Mutation Annotation Format (maf) were analyzed and then used to calculate TMB using the R package “maftools” (18).

#### Testing set

We downloaded three external data sets from the GEO database (https://www.ncbi.nlm.nih.gov/geo/), then eliminated the cases with duplicate or incomplete survival information, and finally got another three validation cohorts, including GSE12276 with 196 samples, GSE21653 with 252 samples and GSE58812 with 107 samples. After removing the batch effects, we merge these three data cohorts into one complete data cohort as our testing set.

### Screening of differentially expressed centrosome-related genes

We collected 726 centrosome-related genes from the database (MiCroKiTS) (http://microkit.biocuckoo.org/) (19) and several associated literature (20–22). The expression information of 699 centrosome-related genes was obtained from the TCGA-BRCA database. The “Deseq2” R package was used to perform differentially expressed genes (DEGs) analysis using raw counts (23). DEGs were determined with a cutoff of an adjust p-value of less than 0.05 and |Log2 fold change| greater than 1.

### Functional enrichment analysis of differentially expressed genes

The “clusterProfiler” (24) R package was used to perform Gene Ontology (GO) and Kyoto Encyclopedia of Genes and Genomes (KEGG) pathway enrichment analysis. With the use of Fisher’s exact test, those with false discovery rate FDR-corrected p-values of less than 0.05 were regarded as marked indicators. Gene set enrichment analysis (GSEA) was performed with the webgestalt (http://www.webgestalt.org/) (25). The “PROGENy” R package was used to assess 14 signaling pathway activities (androgen, estrogen, EGFR, hypoxia, JAK-STAT, MAPK, NFkB, PI3K, p53, TGFb, TNFa, Trail, VEGF and WNT) in patients (26).

### Construction and validation of the centrosome-related prognostic model

First, RNA expression in the TCGA-BRCA, GSE12276, GSE21653 and GSE58812 datasets was cross-checked to identify co-expressed and differentially expressed centrosome-related genes. Consequently, univariate Cox analysis of overall survival (OS) was applied to screen for centrosome-related genes with prognostic value. Subsequently, LASSO regression with 10-fold cross-validation was performed, 1,000 cycles of the “glmnet” R package were run, and 1,000 random stimulations were set (27, 28). Based on the best lambda value, the optimal possible gene was selected to construct the model, and a CRSS was constructed.

The CRSS was calculated based on the expression level of each gene and its corresponding regression coefficients based on the following equation:


CRSS = ∑ genes Cox coefficient × gene expression

The patients were then categorized into the high-risk and low-risk groups according to the best cutoff value, using the R package of “surv_cutpoint” in “survminer”. The predictive sensitivity of the CRSS was painted *via* the R Package “survivalROC” for estimation. The model effectiveness was evaluated in the validation set using the same coefficient and cutoff values that were used in the training set.

### Assessing the immune microenvironment of breast cancer

We used “estimate” algorithm to calculate the stromal and immune scores, as well as the tumor purity of breast cancer samples from TCGA. Then, we used 4 different algorithms, including CIBERSORT, XCELL, QUANTISEQ and TIMER, to estimate the infiltration level of several tumor infiltrating immune cells in tumor immune microenvironment (TIME). In addition, we downloaded the activation levels of the 7-step Cancer Immunity Cycle from the tracking tumor immunophenotype (TIP) (http://biocc.hrbmu.edu.cn/TIP/) (29) and we further described the tumor immune microenvironment through the expression of 11 immune checkpoint genes and immune response scores evaluated by the “easier” algorithm.

### Single-cell transcriptome analysis

We first obtained scRNA-seq data and paired bulk RNA-seq data 24 breast tumors from GEO: GSE176078. We applied each single-cell sample separately to perform unsupervised clustering of the single cells using the read count matrix as input *via* Seurat package (v4.1.1) in R (v4.1.3). The quality control applied to cells was mainly based on the number of detected genes and proportion of mitochondrial gene count per cell. At first, cells with fewer than 200 detected genes and cells with over than 15% mitochondrial gene count were filtered. In order to avoid unexpected noise, genes detected in less than 3 cells were excluded from the downstream analysis. To correct the batch effects, data integration was performed by fast mutual nearest neighbor (fastMNN) method *via* Seurat-Wrappers package (v0.3.0). We next performed dimension reduction clustering and differential expression analysis following the Seurat-guided tutorial. The principal component analysis (PCA) and uniform manifold approximation and projection (UMAP) dimension reduction were conducted with the top 15 principal components.

### Predicting immunotherapy sensitivity and drug responses

To predict the immunotherapy sensitivity, the Immunophenoscore (IPS) was calculated using the Cancer Immunome Atlas (https://tcia.at/). To further validate the predictive value of above immunotherapy responses, we used several extra immunotherapy data sets included GSE123845 (breast cancer), IMvigor210 (uroepithelial carcinoma) and GSE35640 (melanoma) to predict immunotherapy response.

We used the “oncoPredict” R package to assess the predictive ability of CRSS chemotherapeutic agents by calculating patients IC50 for various common chemotherapeutic agents. The Wilcoxon rank test was then used to compare the difference in IC50 between the high/low-risk groups.

### Univariate and multivariable Cox regression

We performed univariate Cox regression on TCGA-BRCA with gene expression and overall survival. Multivariate Cox regression was used to evaluate independent risk factors in the same cohort. Genes and factors with a false discovery rate (FDR)< 0.05 were considered statistically associated with patient survival. The results of univariate and multivariate Cox regression were acquired and visualized by using the R package of “ggforest” in “survminer”.

### Establishment of the nomogram

This study used the Cox regression model along with the R package “rms” to build an OS prediction nomogram that set 1-, 3-, and 5-year OS as the endpoints. The C-index was used to estimate the discriminative ability of the nomogram. Calibration plots were used to visualize the consistency between the predicted and factual 1-, 3-, and 5-year OS.

### Statistical analysis

DEGs were screened using the Wilcoxon test and compared using Fisher’s exact test. Univariate Cox analysis of OS was performed to identify relevant genes and their prognostic value. Kaplan–Meier survival curves were generated and compared between the two groups using the log-rank test. The association between the prognostic model risk score and immune score was assessed using Spearman’s correlation analysis. All statistical analyses were performed using R version 4.1.1 (https://www.r-project.org/) and its adequate packages. Statistical significance was set at p ≤ 0.05.

## Results

### Identification of differentially expressed centrosome-related genes in breast cancer

**Figure 1:**
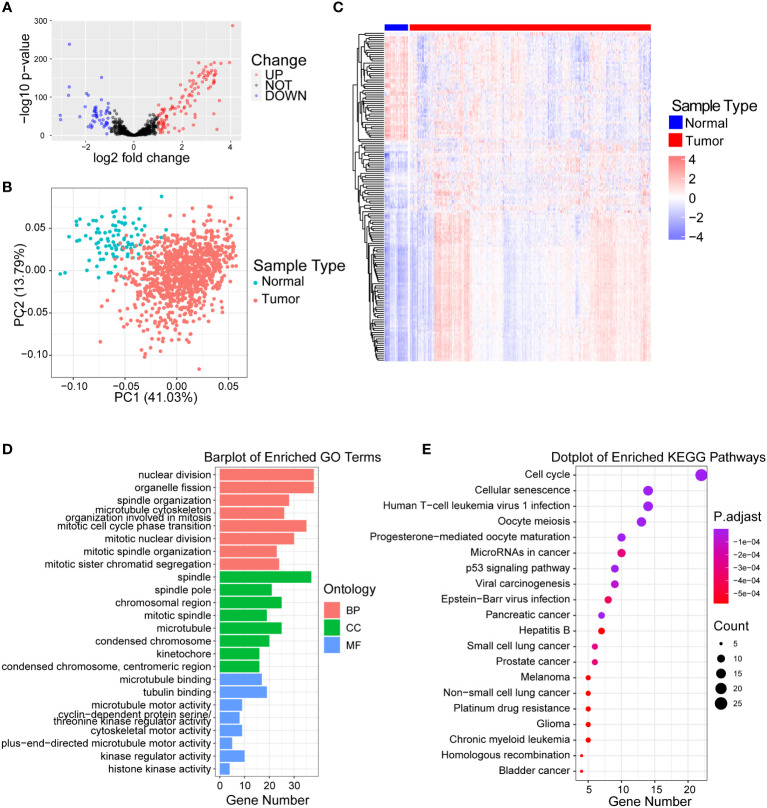
Identification of differentially expressed centrosome-related genes and functional analysis. **(A)** Volcano plot of the differentially expressed centrosome-related DEGs by comparing breast cancer tissues to normal prostate tissues from TCGA-BRCA cohort. Blue represents downregulated genes, and red represents upregulated genes in BRCA. p< 0.05, |log2 fold change| > 1.0. **(B)** Principal component analysis (PCA) of TCGA-BRCA cohort for optimal k = 2. **(C)** Heatmaps of centrosome-related DEGs. **(D)** GO enrichment of upregulated centrosome-related DEGs. **(E)** KEGG pathways of upregulated centrosome-related DEGs.

Through reviewing public databases and literature, we collected a total of 727 centrosome-related genes (**Supplementary Table 1**) and validated that 699 genes were expressed in our training set. Next, we identified 155 centrosome-related DEGs between normal and malignant breast tissues in TCGA-BRCA by applying the Wilcoxon test through the “DESeq2” algorithm. As the volcano plot showed, there were 106 upregulated genes and 49 downregulated genes in the tumor samples compared to normal tissues (**Figure 1A**). Principal component analysis (PCA) analysis showed that the normal and malignant breast tissues could be clearly distinguished by above the 155 differentially expressed CRGs (**Figure 1B**). In addition, the heatmap further revealed significant differences in the expression of these CRG sin normal and malignant breast tissues (**Figure 1C**).

### Functional analysis of centrosome-related DEGs

In order to explore the biological significance of these 155 differentially expressed CRGs between normal and malignant breast tissues, we firstly performed GO analysis on upregulated and downregulated DEGs separately. We found that the upregulated CRGs were significantly enriched in the nuclear division, spindle and cell division terms based on GO enrichment analysis (**Figure 1D**; **Supplementary Table 2**). Additionally, we found that the pathways such as cellular responses to peptides, proteasome activity were enriched in downregulated CRGs (**Figure S1A**; **Supplementary Table 2**). Similarly, KEGG analysis revealed that upregulated centrosome-related DEGs in tumor tissues were mainly enriched in cell cycle, cellular senescence and p53 signaling pathway (**Figure 1E**; **Supplementary Table 2**), while downregulated CRGs were enriched in signaling pathways like sphingomyelin and prolactin (**Figure S1B**; **Supplementary Table 2**). The significant enrichment of cell cycle and mitotic pathways in malignant breast tissues illustrates the important role of centrosomes during breast cancer development (30, 31). It also verified that these differentially expressed CRGs could discriminate the difference in biological function between normal and malignant breast tissues.

### Establishment of a centrosome-related prognostic model for breast cancer

**Figure 2:**
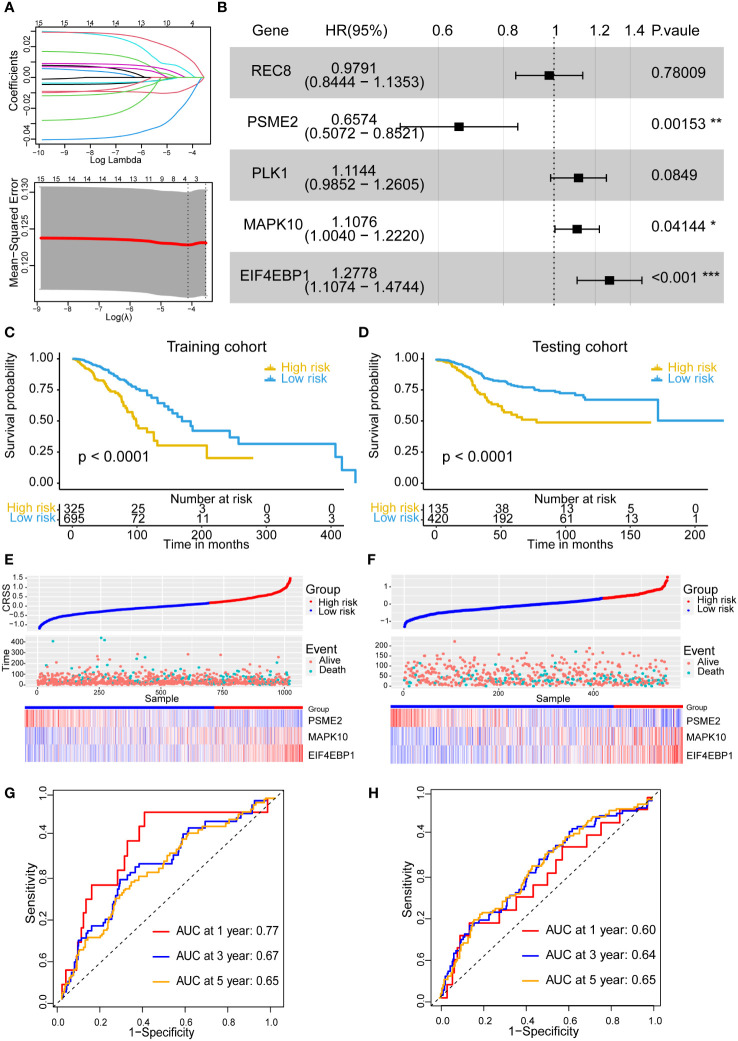
Establishment and stability validation of centrosome-related prognostic model. **(A)** The Least Absolute Shrinkage and Selection Operator (LASSO) Cox regression for the centrosome-related prognostic DEGs. **(B)** The multivariable Cox regression analysis of five genes based on cross-validation and the minimum partial likelihood deviance to further demonstrate the independent prognosis-related genes and obtain the genes index. **(C)** Kaplan–Meier analysis for OS curves of patients from TCGA-BRCA in high/low-risk subgroups in training cohort. **(D)** Kaplan–Meier analysis for OS curves of patients from GEO in high/low-risk subgroups in validation cohort. **(E)** Distribution of CRSS and patterns of the survival time and survival status between the high/low-risk subgroups for the training set. **(F)** Distribution of CRSS and patterns of the survival time and survival status between the high/low-risk subgroups for the validation set. **(G)** Time-related ROC analysis exhibited the prognostic value of the CRSS in the training set. **(H)** Time-related ROC analysis exhibited the prognostic value of the CRSS in the validation set. The asterisks represent the statistical P value (*p < 0.05; **p < 0.01; ***p < .001).

By univariate Cox regression analysis, we selected 15 genes significantly associated with breast cancer patients prognoses (P< 0.05) from 155 centrosome-related DEGs, including *STAT5A, REC8, RAD51, PSME2, PLK1, MAPK10, HAP1, EIF4EBP1, DONSON, DCX, CDH13, CCNE1, CCNE2* and *CCND2*. The Cox-LASSO regression algorithm was then performed to identify genes with the most robust prognostic value. Tenfold cross-validation was applied to overcome overfitting, with an optimal λ value of 0.012 selected (**Figure 2A**). Eventually, three candidate genes (*MAPK10*, *EIF4EBP1*, *PSME2*) were identified as independent prognostic genes and were included in the centrosome-related prognostic model (**Figure 2B**). Therefore, we computed the centrosome-related signature score (CRSS) based on the expression levels of these three candidate genes: CRSS = MAPK10*0.116+EIF4EBP1*0.305-PSME2*0.443

### Prognostic analysis of the centrosome-related prognostic model

Based on the best cutoff value, 325 and 695 patients were categorized into the high- and low-risk groups, respectively. Prognostic analysis showed that the high-risk group had an obviously poor prognosis (p< 0.0001) (**Figure 2C**). The number of deaths in the high-risk group was significantly higher than that in the low-risk group (**Figure 2E**). The area under the curve (AUC) for predicting 1-, 3-, and 5-year OS was 0.77, 0.67, and 0.65, respectively (**Figure 2G**).

We further analyzed the association of CRSS with clinical manifestations and pathological features of breast cancer patients. **Table 1** obviously indicated that CRSS was indistinguishable from clinical information and pathological features. Multifactorial Cox regression analysis also confirmed that CRSS can be used as an independent prognostic tool for breast cancer patients (**Figure S1C**).

**Table 1:** Clinicopathological associations of CRSS in breast cancer.

Variables	Low risk	High risk	P. value
Age at diagnosis, years			**<0.0001**
≤50	232	238	
>50	463	87	
Stage			0.3221
I+II	590	266	
III+IV	104	57	
ER			**<0.0001**
Negative	111	108	
Positive	555	203	
PR			**<0.0001**
Negative	164	147	
Positive	501	163	
HER2			0.9781
Negative	357	162	
Positive	104	46	
Survival state			**<0.0001**
Death	77	69	
Alive	618	256	

Currently, the diagnosis and treatment of breast cancer is guided mainly based on the clinical and pathological staging (32). However, the patients prognoses in the same period varies somewhat, so we further explored the prognostic status of patients under different stages. Our results showed that CRSS could accurately predict the prognosis of stage II and III patients, and patients with high CRSS showed a worse prognosis (stage II: p=0.0038; stage III: p<0.0001) (**Figures S2B**, **C**). However, it failed to distinguish the prognosis of breast cancer patients with stage I and stage IV (**Figures S2A, D**). We suspected that this might be owing to the small number of stage I and stage IV breast cancer patients, and our increasing awareness of early prevention of breast cancer. In addition, CRSS enabled prognostic prediction for patients with different molecular subtypes (LumA, LumB, Her2, Basal). Patients with high CRSS showed worse prognosis in all molecular subtypes, and notably the predictive power of CRSS was more significant in patients with LumB and Her2. (LumA: p=0.088; LumB: p=0.018; Her2: p=0.00056; Basal: p=0.088) (**Figures S2E–H**).

We then devoted to use additional independent datasets to validate the prognostic predictive ability of the prognostic model. After excluding cases with duplicate or incomplete survival information and correcting the batch effects, we merged three data sets including GSE12276 with 196 samples, GSE21653 with 252 samples and GSE58812 with 107 samples into a new validation cohort (555 samples). Likewise, patients in the validation cohorts were also divided into the high- and low-risk groups according to the same cutoff value and prognostic model as those in our training cohort. In the validation cohort, we obtained the similar results. The high-risk group exhibited poor prognostic performances (p< 0.0001) and more deaths (**Figures 2D, F, H**). These results demonstrated that the centrosome-related prognostic model based on these three candidate genes (*MAPK10*, *EIF4EBP1*, *PSME2*) shows high accuracy and considerable stability in predicting the prognoses of breast cancers.

### Functional analysis of the centrosome-related prognostic model

**Figure 3:**
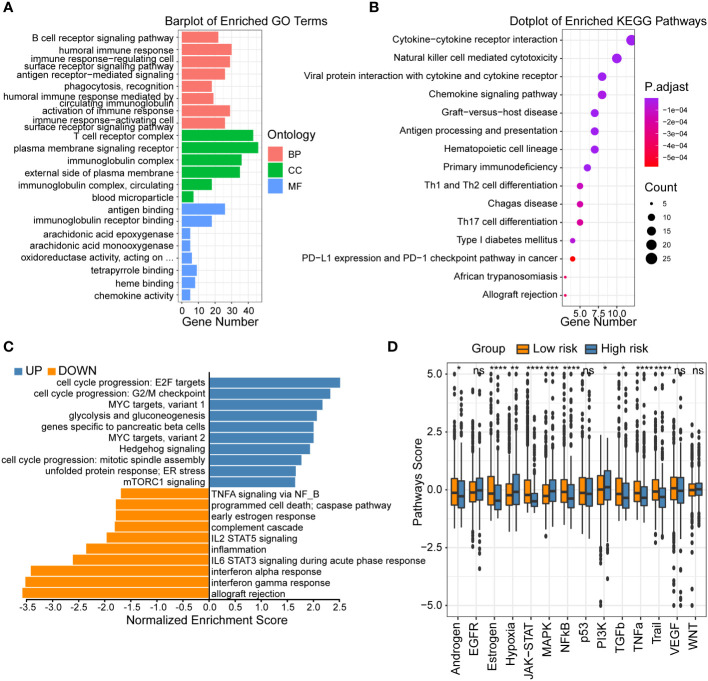
Functional analysis of centrosome-related prognostic model. **(A)** GO enrichment of centrosome-related DEGs in low-risk subgroups. **(B)** KEGG pathways of centrosome-related DEGs in low-risk subgroups. **(C)** Gene set enrichment analysis (GSEA) of centrosome-related prognostic model. **(D)** Analysis of 14 cancer-related pathway activities score. The asterisks represent the statistical P value (*p < 0.05; **p < 0.01; ***p < .001; ****p < 0.0001; ns p > 0.05).

To investigate the underlying mechanisms that led to the different outcome stratified by CRSS, we first performed GO as well as KEGG analysis based on the DEGs between the high- and low-risk group patients. The GO and KEGG enrichment analyses revealed that axon development, synaptic membrane and calcium signaling pathway were upregulated in the high-risk group (**Figures S2A, B**; **Supplementary Table 2**). However, the low-risk group patients mainly showed enrichment in immune responses, such as humoral immune response, T cell receptor complex, natural killer cell mediated cytotoxicity, antigen processing and presentation (**Figures 3A, B**; **Supplementary Table 2**). Furthermore, the GSEA analysis showed that the gene sets involved in interferon gamma/alpha response and inflammation were gathered together in low-risk group patients. It has been reported that MYC targets (33) and Hedgehog signaling (34) are more active in tumor tissues. In contrast, signaling pathways including cell cycle progression, MYC targets and Hedgehog signaling were enriched in high-risk group patients (**Figure 3C**).

To further compare the molecular functional differences across high/low-risk patients, we calculated 14 cancer-related pathway activities. Hypoxia leading to angiogenesis and metabolic rewiring is a recognized driver of breast cancer aggressiveness, treatment resistance, and poor prognosis (35). Our results showed that high-risk group patients had higher hypoxia signature score (**Figure 3D**). We also found high-risk group patients revealed upregulated levels of glycolysis and gluconeogenesis, which represented the breakdown of glucose or glycogen to lactate in the presence of insufficient oxygen (36). RTK is a class of cell surface receptors that responds to environmental signals by initiating appropriate signaling cascades in tumor cells and regulates various downstream signaling pathways including MAPK, PI3K/Akt, etc. (37) Our results showed no significant difference in RTK (EGFR, VGFR) activity between patients in different groups, but its downstream signaling pathway (MAPK, PI3K/Akt) was higher in high-risk group patients. Tumor necrosis factor-related apoptosis-inducing ligand (TRAIL) induces cancer cell regulation without causing toxicity in mice has received much attention (38). Our findings exhibited that low-risk group patients harbored higher TRAIL signature score, suggesting a higher apoptotic potential. In summary, the high- and low-risk patients exhibited distinct functional activities in immune responses, inflammation, and other oncology-related signaling pathways. These results could partly explain the underlying mechanisms how this prognostic model was used to assess the prognoses of breast cancer patients.

### Comparison of the mutation profiles in high/low-risk breast cancer patients

**Figure 4:**
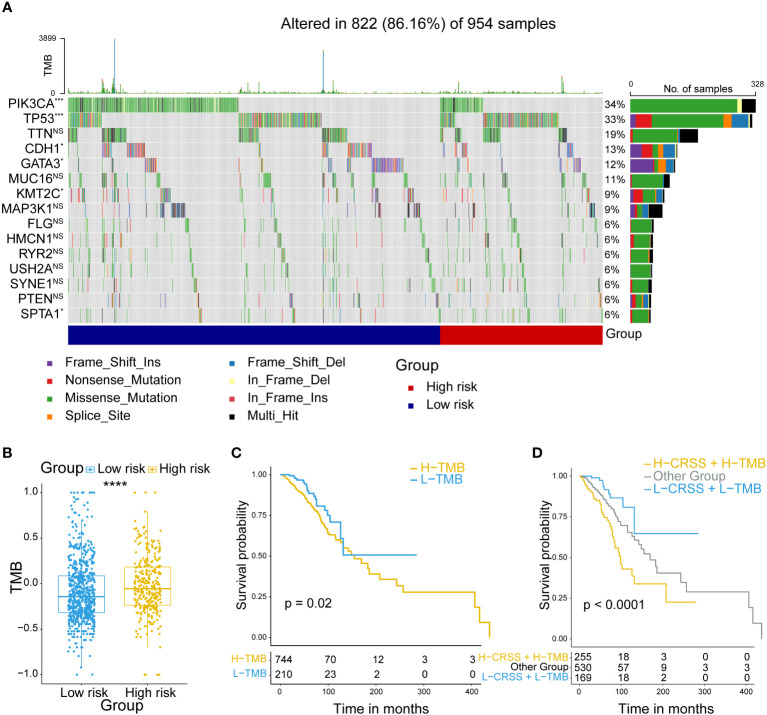
Mutation analysis of centrosome-related prognostic model. **(A)** Comparison of the mutation landscape between groups with high/low-risk. **(B)** Tumor mutant burden (TMB) difference among groups with high/low-risk. **(C)** Kaplan-Meier analyses of OS in breast cancer patients, stratified according to TMB values. **(D)** Kaplan-Meier analyses of OS in breast cancer patients stratified according to the combination of CRSS and TMB. The asterisks represent the statistical P value (****p < 0.0001).

Multiple researches have shown that the mutational pattern plays an important role in tumor development (39). Therefore, we next compared the mutational landscapes between different risk group patients. *PIK3CA*, *TP53*, *CDH1* and *GATA3* which were confirmed as somatic driver substitutions and small insertions/deletions (indels), were previously reported to be implicated in breast cancer development (40). Our results showed that they exhibited still significantly different mutation rates in high/low-risk groups (**Figure 4A**). To evaluate the level of tumor mutation burden (TMB) which was the best quantitative criterion to reflect the mutation level, we calculated the TMB score for every patient, and found that the high-risk group patients had higher TMB score (**Figure 4B**). Next, we performed a prognostic analysis to figure out the prognostic value of the TMB level. The results showed that the TMB level was associated with an unfavorable outcome (**Figure 4C**). Considering the possible synergistic effect of TMB and CRSS on prognosis, we performed a novel stratified prognostic analysis and by combining TMB and CRSS. Surprisingly, we found an improvement for survival prediction within the training cohort. The results showed that patients with high CRSS and TMB score were robustly linked with an inferior prognosis, while patients with low CRSS and TMB score had an improved prognosis (P<0.0001) (**Figure 4D**). Together, these data demonstrated that higher frequency of somatic mutations occurred in high-risk group patients, and the combination of CRSS and TMB could further refine the prognostic prediction of breast cancer patients.

### Diverse tumor immune microenvironment components among high/low-risk patients

When exploring the differences of biological functions in different group patients, we found that the immune system might have a considerable connection with our centrosome-related prognostic model. Thus, we further investigated the tumor immune microenvironment (TIME) which acted as an important role in tumorigenesis and therapy responses (41).

**Figure 5:**
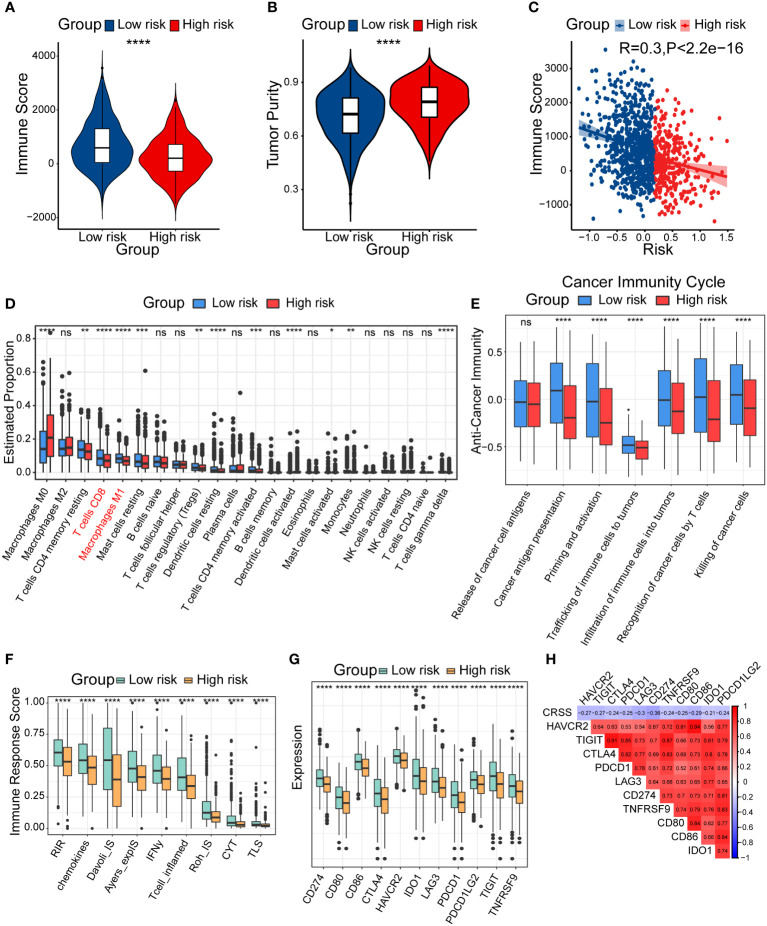
Diverse tumor immune microenvironments among high/low-risk patients. **(A)** Immune score in high/low-risk subgroups. **(B)** Tumor purity in high/low-risk subgroups. **(C)** Spearman correlation between Immune score and CRSS. **(D)** The differential estimated proportion of 22 CIBERSORT immune cell types in high/low-risk subgroups. The central line represents the median value. The bottom and top of the boxes are the 25th and 75th percentiles (interquartile range). The whiskers encompass 1.5 times the interquartile range. **(E)** Activities of seven-step cancer immune cycle. **(F)** Immune response score in high/low-risk groups. **(G)** The expression of immune-related checkpoints genes among high/low-risk subgroups. **(H)** Correlations between CRSS and expression of immune-related checkpoints genes. The asterisks represent the statistical P value (*p < 0.05; **p < 0.01; ***p < .001; ****p < 0.0001; ns p > 0.05).

Firstly, we evaluated the immune score and tumor purity in different risk groups and calculated the correlation between CRSS and immune score by ESTIMATE algorithm. The results showed that lower immune score but higher tumor purity was found in the high-risk group patients (**Figures 5A, B**). We also discovered a significant negative correlation between CRSS and immune score (**Figure 5C**). Therefore, we speculate that the high-risk group had a poor prognosis probably because of the restricted immune cell infiltration.

To further clarify the diverse immune landscapes, we explored the distribution of 22 immune cells in the TIME among breast cancer patients by conducting the CIBERSORT algorithm. The results revealed that the abundance of M1 macrophages, CD8^+^ T cells and resting memory CD4^+^ T cells were significantly lower in the high-risk group (**Figure 5D**). We also calculated the immune cell infiltration using other additional algorithms including XCELL, QUANTISEQ and TIMER (**Figures S3A–C**), and found similar results with CIBERSORT. These results suggested that high/low-risk patients had distinct abundance of diverse immune subsets and potentially different anti-tumor capacity.

In addition, some scholars proposed a seven-step immune process in 2013, which was called the cancer immune cycle (42). Anti-tumor immunity need begin, develop and expand this series of events to be effective to kill cancer cells. Apparently, several processes including the step2 (cancer antigen presentation), step5 (infiltration of immune cells into tumors), step6 (recognition of cancer cells by T cells), and step7 (killing of cancer cells) were significantly lower in the high-risk group (**Figure 5E**). To further explore the distinct immune responses between patients in different risk groups, we assessed immune response score using the easier algorithm. We found that the high-risk group showed lower levels of a variety of immune responses, such as RIR, chemokines, T cell inflamed, which further confirmed our speculation about the immunosuppressive status of the high-risk group patients (**Figure 5F**).

The expression of immune checkpoint genes has been reported to be lower in non-inflammatory TIME (43). We consistently found lower expression of most immune checkpoint genes in the high-risk group, including *CD274*, *CD80*, *CD86*, *CTLA4*, *HAVCR2*, *IDO1*, *LAG3*, *PDCD1*, *PDCD1LG2*, *TIGIT* and *TNFRSF9*. Correlation analysis showed that CRSS was negatively correlated with the expression of immune checkpoint genes (**Figures 5G, H**). In summary, patients in diverse risk groups showed significant differences in immune cell infiltration and tumor immune cycles in TIME. Particularly, the high-risk group patients exhibited an immune-desert and immunosuppressive phenotype.

### Distinct cellular composition of high/low-risk breast cancer patients at single-cell resolution

**Figure 6:**
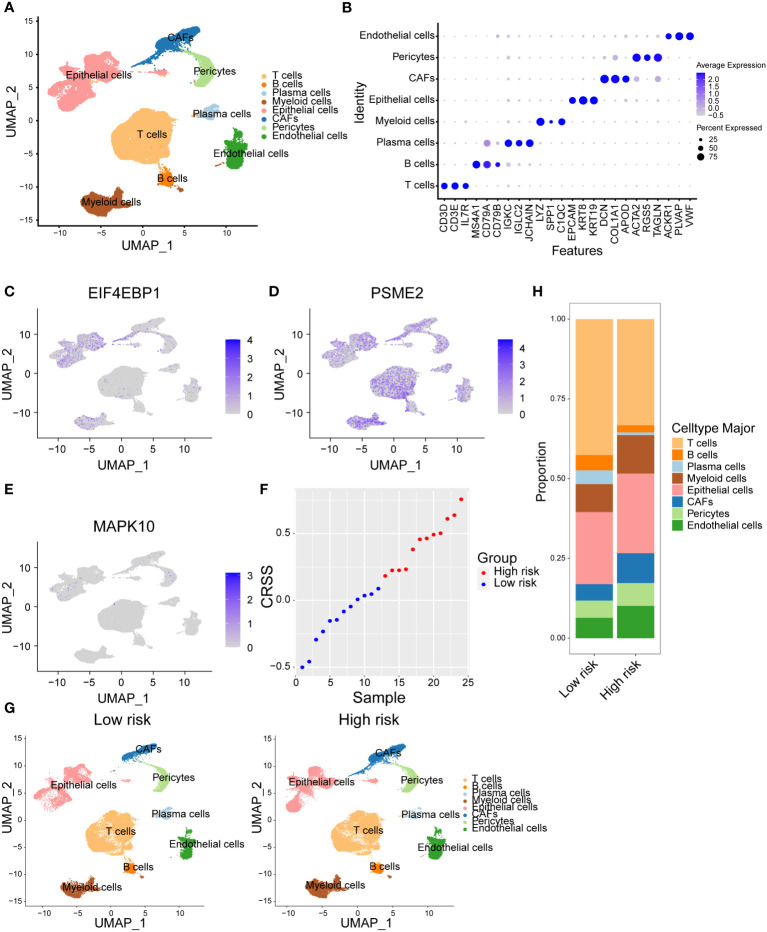
Distinct cellular composition of high/low-risk breast cancer patients at single-cell resolution. **(A)** Cells were clustered into eight types *via* tSNE dimensionality reduction algorithm, each color represented the annotated phenotype of each cluster. **(B)** Dot plot of the top three marker genes expression of each cluster. **(C–E)** Expression of three centrosome-related prognostic genes in each cluster. **(F)** CRSS of 24 samples from breast cancer single cell dataset GSE176078. **(G)** eight cell clusters in the high/low-risk groups were identified *via* tSNE dimensionality reduction algorithm. **(H)** The proportion of cells in high/low-risk groups of breast cancer single cell dataset GSE176078.

In order to figure out the different cellular landscape of breast cancer patients in high-risk and low-risk groups, we integrated 24 published single-cell RNA-seq data with paired bulk RNA-seq data of breast cancer samples. After strict quality control within every single sample and batch effects correction among diverse samples *via* fastMNN function, we visualized the high-resolution transcriptional atlas of breast cancer by uniform manifold approximation and projection (UMAP) (**Figure 6A**). Next, we annotated epithelial cells, endothelial cells, pericytes, cancer-associated fibroblasts (CAFs), myeloid cells, T cells, B cells and plasma cells based on the canonical cell markers (**Figures 6A, B**). For example, we identified epithelial cell subsets based on the expression level of *EPCAM*, *KRT18* and *KRT19*. In addition, T cells were defined owing to the expression of *CD3D*, *CD3E* and *IL7R* (**Figure 6B**). Next, we aimed to decipher which cellular subsets expressing the three candidate genes in our risk model. As shown in the UMAP plot, *EIF4EBP1* was mainly overexpressed in the epithelial cells and weakly expressed in myeloid cells (**Figure 6C**). Additionally, *PSME2* was universally expressed in every subset, particularly T and myeloid cells (**Figure 6D**). Moreover, *MAPK10* was only sparsely expressed in epithelial cells and pericytes (**Figure 6E**). Considering these 24 published single-cell samples had paired bulk RNA-seq data, we calculated the CRSS of each sample based on our risk model formula. Therefore, we divided the samples into high-risk and low-risk groups based on the median value of CRSS (**Figure 6F**). These two risk groups revealed different distributions in the UMAP plot (**Figure 6G**). The composition of diverse cellular subpopulations in high/low-risk group was compared. We found that the composition of lymphocytes including T cells, B cells and plasma cells were significantly lower in the high-risk group. However, the immunosuppressive myeloid cells showed a higher frequency in the high-risk group (**Figure 6H**). Taken together, we characterized the expression landscape of the candidate centrosome-related markers in our risk model and revealed distinct composition of immune subsets in high/low-risk group breast cancer patients.

### Diverse treatment responses in high/low-risk group patients

**Figure 7:**
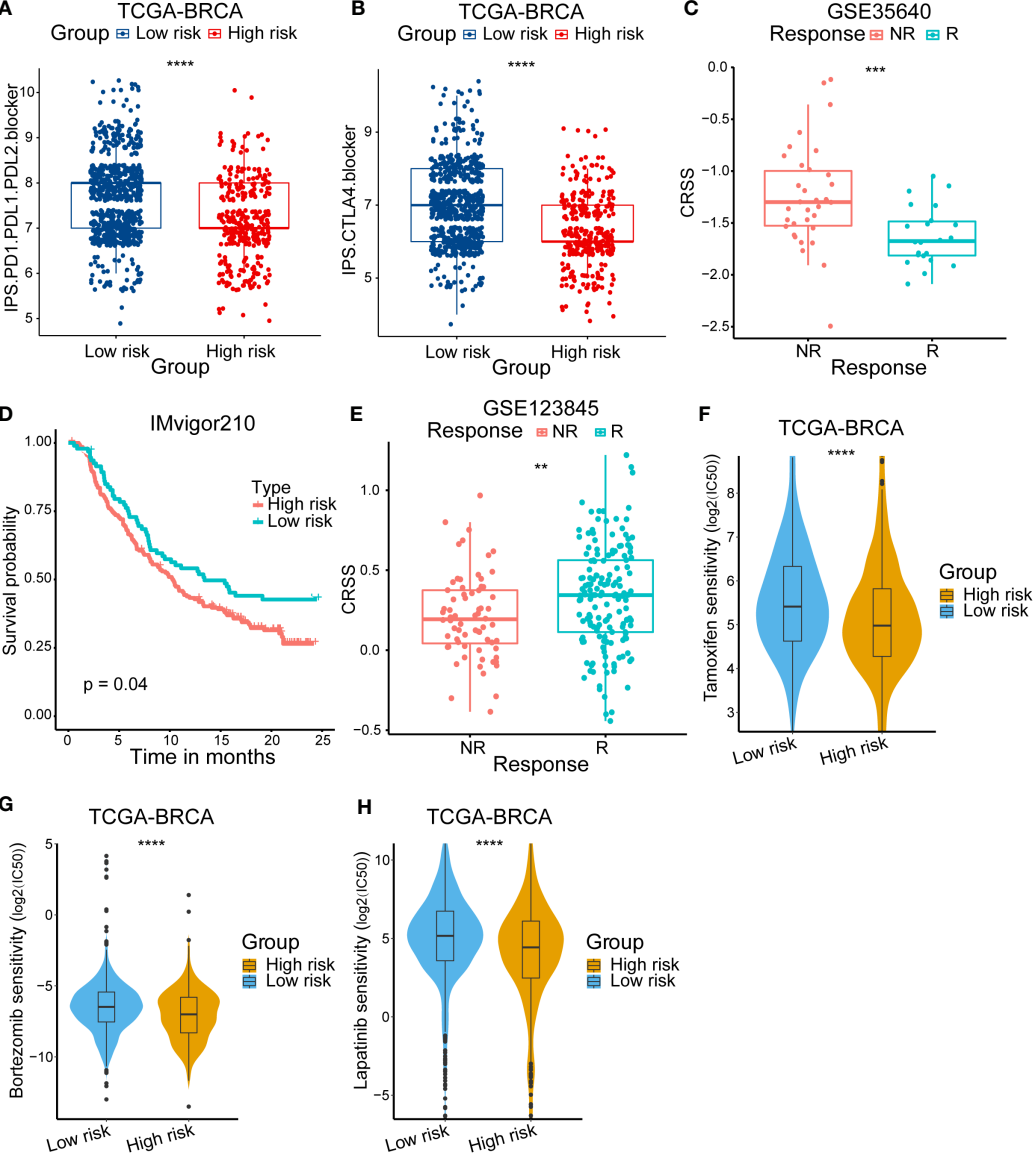
Validation of immunotherapy response and the difference of anticancer drug sensitivity. **(A, B)** Immunophenoscore (IPS) score in high/low-risk subgroups. **(C)** Kaplan–Meier survival curve of the patients in high/low-risk groups for OS in the PD-1/PD-L1 treatment cohort (IMvigor210). **(D)** Analysis of the immunotherapy response between high/low-risk groups in the high-grade melanoma immunotherapy cohort (GSE35640). **(E)** Analysis of the neoadjuvant chemotherapy between high/low-risk group in breast cancer cohort (GSE123845). **(F)** The IC50 of Tamoxifen among high/low-risk groups. **(G)**The IC50 of Bortezomib among high/low-risk groups. **(H)** The IC50 of Lapatinib among high/low-risk groups. The asterisks represent the statistical P value (**p < 0.01; ***p < .001; ****p < 0.0001).

Considering the differences of immune status within different risk group patients, we then compared the response to anti-CTLA4/PD-1/PD-L1 therapy. The results showed that the low-risk group patients were sensitive to anti-CTLA4/PD-1/PD-L1 therapy (**Figures 7A, B**). We also used other datasets to validate the stability of our results. In the high-grade melanoma cohort (GSE35640), CRSS was higher in patients who failed to respond to immunotherapy (**Figure 7C**). Additionally, in the uroepithelial tumor cohort (IMvigor210), patients with high CRSS had a worse prognostic presentation (**Figure 7D**).

Then, we devoted to figure out if high/low-risk patients were sensitive to neoadjuvant chemotherapy. The GSE123845 cohort included 210 invasive breast cancer patients who received a standard neoadjuvant chemotherapy (NAC). We found that patients responding to NAC had a higher CRSS (**Figure 7E**). In addition, we also compared the sensitivity to diverse anti-cancer chemotherapy drugs between different group patients. We calculated the IC50 for several drugs which were commonly used to treat breast cancer according to the National Comprehensive Cancer Network (https://www.nccn.org/) guidelines. A series of drugs were found to have a lower IC50 in high-risk patients such as tamoxifen, bortezomib and lapatinib (**Figures 7F–H**). In summary, these results illustrated that high-risk patients may be insensitive to immunotherapy but sensitive to NAC and a set of anti-cancer drugs, which may guide the treatment choice of breast cancer patients.

### Construction and validation of a centrosome-related prognostic model associated nomogram

**Figure 8:**
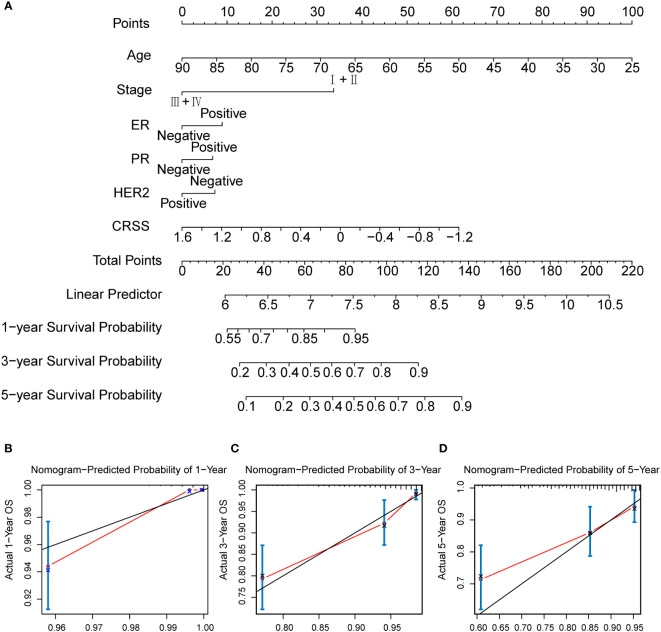
The calculation of centrosome-related prognostic nomogram. **(A)** The prognostic nomogram to predict the 1-, 3-, and 5-year OS of breast cancer patients. For each patient, we calculated the points of the clinical–pathological features and summed up the points to obtain the total points. The predicted 1-, 3-, and 5-year OS can be estimated based on the total points of each patient. **(B)** The calibration curves for predicting patient survival at 1-year OS. **(C)** The calibration curves for predicting patient survival at 3-year OS. **(D)** The calibration curves for predicting patient survival at 5-year OS.

To further investigate the value of the CRSS for clinical application, we examined the CRSS as an independent clinical indicator along with other clinical information. As previously described, CRSS, age, stage, and hormone receptor expression were all the independent prognostic risk factor for breast cancer patients (**Figure S1C**). Therefore, we combined the CRSS with other clinical information of independent predictors suggested by multivariate Cox analysis to construct a nomogram. In the nomogram, the CRSS contributed significantly to the prediction of survival probability (**Figure 8A**). As shown in the calibration curve, the 1-, 3-, and 5-OS predicted by the column line plot was generally consistent with the actual OS (**Figures 8B–D**), indicating that the column line plot had great reliability and value in clinical applications.

## Discussion

So far, breast cancer remains one of the most common and fatal malignancies in women worldwide (1, 2). As a highly heterogeneous disease, breast cancer exhibits extremely distinct outcomes among individuals (44, 45). The precise prognosis and treatment of breast cancer are far from satisfying. Therefore, it is imperative to develop a novel tool for the stratification of breast cancer patients. In recent years, studies concerning genomics and transcriptome as well as single-cell multi-omics data have greatly contributed to our understanding of breast cancer. However, most of these studies are mainly based on cell functions and certain molecular, there are few researches focusing on the role of certain subcellular structures.

The centrosome contains a pair of centrioles at its core. With a ninefold symmetric structure, the centrioles are embedded in its surrounding matter and form a complete centrosome (46). The centrosome acts as the main microtubule-nucleating organelle in animal cells and plays a critical role in mitotic spindle orientation and in genome stability (47). During the G1 phase of the cell cycle, the separation of the two centrioles of the old centrosome is allowed to replicate, and at the end of the replication process, each of the two old centrioles combines with a new centriole to become a new centrosome (48). The network of centrosomes involved in actin and tubulin interactions and regulation plays a crucial role in cell dynamics and cell polarity. Centrosome amplification, instability and mis-regulation have been shown to be essential factors in carcinogenesis (49). During an entire cell cycle, each single action of the centrosome is regulated by genomic and cytokine regulation. To our knowledge, this study is the first prognostic model based on a centrosome-related gene set. In this study, we developed and validated a novel prognostic model, CRSS, which independently predicts the prognosis of breast cancer patients. This may provide a precise method to evaluate prognosis and guide treatment in breast cancer patients.

In our study, we extracted 155 differentially expressed CRGs between normal and malignant breast tissues by DESeq2. Univariate Cox regression analysis and the LASSO algorithm were used to identify candidate genes for the centrosome-related prognostic model, including *MAPK10*, *PSME2*, *EIF4EBP1*. *MAPK10* is a member of the MAP kinase family and is activated by threonine and tyrosine phosphorylation (50). The MAP kinase signaling cascade response is regulated by multiple cellular factors, and small changes in it can profoundly affect centriole assembly and cell cycle fidelity (51). *PSME2* is mainly associated with the assembly of cellular pre-replication complexes (52). *EIF4EBP1* is the recipient of our focus and it is a member of a family of encoded translation blocking proteins that interact with a variety of protein kinases such as *CDK1*, *CDK12*, *PLK1* (53). Phosphorylated *EIF4EBP1* regulates eukaryotic mitosis as a translation regulator, which may be important in tumorigenesis and mitotic centrosome function (54). Subsequently, we evaluated the prognostic value of this model in the TCGA training set and in three independent external validation sets. According to CRSS, breast cancer patients were stratified as high/low-risk group. We analyzed the clinicopathological characteristics of patients in different risk groups and explored the differences in the mutational landscape and tumor immune microenvironment of patients. We found that the upregulated genes in the high-risk group patients were associated with tumor signaling pathway, such as focal adhesion and ErbB signaling pathway. Moreover, higher level of TMB score was found in the high-risk group patients. In addition, high-risk group patients also exhibited a suppressive immune microenvironment based on the results of several deconvolution algorithms and single-cell RNA-seq analyses. Next, we explored how patients responded to diverse treatments and tried to offer different recommendations based on CRSS risk groups. The findings revealed that high-risk group patients were resistant to immunotherapy but sensitive to chemotherapy, specially several anti-cancer drugs. Finally, we constructed a nomogram based on CRSS and several clinicopathological features as a way to quantify the risk assessment and survival probability of breast cancer patients.

In breast cancer, estrogen receptor (ER), progesterone receptor (PR) and human epidermal growth factor receptor-2 (HER2) are the main factors that determine the pathological type and treatment options (55, 56). CRSS was differentially expressed in various ER^+/-^ and PR^+/-^ patients, but not in HER2^+/-^ patients. Overall, high-risk group breast cancer patients showed worse survival prognoses. Additionally, CRSS was also a good predictor of prognosis in breast cancer patients among different molecular subtypes (LumA, LumB, Her2, Basal) and clinical stages.

For example, The accumulation of mutations of multiple genes contributes to the tumorigenesis of breast cancer (39). Accumulating studies have shown that genes such as *PIK3CA*, *TP53*, and *CDH1* are among the most frequently mutated genes in breast cancer and are directly or indirectly responsible for breast cancer development and progression. Besides, *PIK3CA* and *TP53* have been reported to be implicated in cancer immunotherapy. Immune checkpoint inhibitors (ICBs) are an important class of anti-cancer therapeutics that block the T-cell inhibitory molecules PD-1 and PD-L1, while TMB levels may reflect the potential for immunogenicity and are associated with the response to ICBs (41, 57, 58). We detected differences in mutations in *PIK3CA*, *TP53*, *CDH1* and *GATA3* between the different risk groups, and an elevated level of TMB score in the high-risk group. These data demonstrated that high- and low-risk group patients had distinct mutational landscape.

Several researches have reported that centrosomes are closely related to immunity, for example, centrosome defects can promote immune escape of tumor cells (12). Therefore, we focused on investigating the differences in immune cell subset composition between the different risk groups. The tumor microenvironment (TME) is mainly composed of tumor cells and non-tumor cells during tumor growth, which reveals a dynamic balance in the process of tumorigenesis, growth and metastasis (59). Immune cells exert a specific role in the TME, where they form an independent TIME that influences cancer progression and treatment response. As the basis of anti-tumor therapy, T cells are an important component of tumor infiltrating immune cells (60). Studies have shown that infiltrating CD8^+^ T cells are a favorable factor for immunotherapeutic response. Tumor-associated macrophages (TAM) have been shown to directly and indirectly regulate PD-1/PD-L1 expression in the tumor environment, where M1-type macrophages have been shown to have anti-tumor effects (61). Therefore, understanding the TME characteristics of breast cancer is a promising approach to improve the response rate of breast cancer immunotherapy. In our centrosome-related prognostic model, patients in the high-risk group exhibited a suppressive immune microenvironment with low infiltration of M1 macrophages and CD8^+^ T cells, implying that patients in the high-risk group had a lower cytotoxic capacity. In addition, enrichment analysis and anti-cancer immune circulation indicated that patients in the high-risk group were under an immunosuppressive state. Besides, we further confirmed that patients in the high-risk group harbored lower composition of lymphocytes at single-cell resolution based on the public single-cell dataset, especially T and B lymphocytes. These data may shed light on the reasons for the poor prognosis of patients in the high-risk group.

Given the heterogeneity of the immune status of patients in different risk groups, we further analyzed the response of these patients to immunotherapy. We found that patients in the high-risk group were resistant to anti-CTLA4/PD-1/PD-L1 therapy, and we also validated the stability of CRSS-predicted immunotherapy in additional immunotherapy datasets, including urothelial tumors (IMvigor210) and high-grade melanoma immunotherapy cohort (GSE35640), all of which illustrated that the high-risk group was resistant to anti-cancer immunotherapy. We also found that patients who responded to NAC had a higher CRSS in the breast cancer cohort (GSE123845). Furthermore, we calculated the IC50 of multiple common breast cancer chemotherapy drugs such as tamoxifen, bortezomib, and we found that patients in the high-risk group may be more sensitive to these chemotherapy drugs.

Undeniably, there are still some limitations of our study. First, this study lacks validation of laboratory data and clinical data, so the value of centrosome-related prognostic models and the assessment of clinical applicability possibilities need to be further validated in larger prospective trials. Second, further experimental studies are needed to elucidate the biological functions of these 3 genes. Finally, because of the significant immunological and clinical prognostic differences between patients with metastatic and non-metastatic breast cancer, further consideration of the impact of metastasis is required.

In summary, we developed a novel centrosome-related prognostic model to predict the prognosis of breast cancer patients, which provides a new reference for breast cancer treatment. According to our prediction model, patients with low expression levels of protective genes and high expression levels of risk genes will obtain higher CRSS, and patients in the high-risk group were resistant to immunotherapy. In addition, we predict that some chemotherapeutic agents may be sensitive in the high-risk group. Combining this information with clinicopathological features, we constructed a column line graph to quantify the risk assessment of individual patients. The centrosome-related prognostic model may be a practical tool to select high- and low-risk patients who may benefit from diverse treatments, thus facilitating personalized management of breast cancer.

## Data availability statement

The datasets presented in this study can be found in online repositories. The names of the repository/repositories and accession number(s) can be found in the article/**Supplementary Material**.

## Author contributions

ZF and Z-JG are responsible for collating documents and performing these experiments. XY gave the experimental guidance. ZF and Z-JG are responsible for writing this article, while S-RS and FY are responsible for proofreading and submission. All authors contributed to the article and approved the submitted version.

## Supplementary material

Supplementary Figure 1 Functional analysis of centrosome-related DEGs and multivariate Cox regression analysis of forest plots **(A)** GO enrichment of downregulated centrosome-related DEGs. **(B)** KEGG pathways of downregulated centrosome-related DEGs. **(C)** Multivariate Cox regression analysis of prognostic factors based on cross-validation and least partial likelihood deviation further argued for independent prognostic factors.

Supplementary Figure 2 Subgroup survival analysis and functional analysis between high/low-risk groups **(A)** Kaplan–Meier survival for OS curve of the patients in the high/low-risk groups in stage I breast cancer patients. **(B)** Kaplan–Meier survival for OS curve of the patients in the high/low-risk groups in stage II breast cancer patients. **(C)** Kaplan–Meier survival for OS curve of the patients in the high/low-risk groups in stage III breast cancer patients. **(D)** Kaplan–Meier survival for OS curve of the patients in the high/low-risk groups in stage IV breast cancer patients. **(E)** Kaplan–Meier survival for OS curve of the patients in the high/low-risk groups in LumA breast cancer patients. **(F)** Kaplan–Meier survival for OS curve of the patients in the high/low-risk groups in LumB breast cancer patients. **(G)** Kaplan–Meier survival for OS curve of the patients in the high/low-risk groups in Her2 breast cancer patients. **(H)** Kaplan–Meier survival for OS curve of the patients in the high/low-risk groups in Basal breast cancer patients. **(I)** GO enrichment of centrosome-related DEGs in high-risk subgroup. **(J)** KEGG pathways of centrosome-related DEGs in high-risk subgroup.

Supplementary Figure 3 Immune cell infiltration in high/low-risk subgroups. **(A)** Different estimated proportions of XCELL immune cell types in high/low-risk subgroups. **(B)** Different estimated proportions of TIMER immune cell types in high/low-risk subgroups. **(C)** Different estimated proportions of QUANTISEQ immune cell types in high/low-risk subgroups
