# Supplementary figures and images for: Identification of a centrosome-related prognostic signature for breast cancer
Source: Front Oncol. 2023 Mar 22;13:1138049. doi: 10.3389/fonc.2023.1138049 (PMC10073657; doi:10.3389/fonc.2023.1138049)

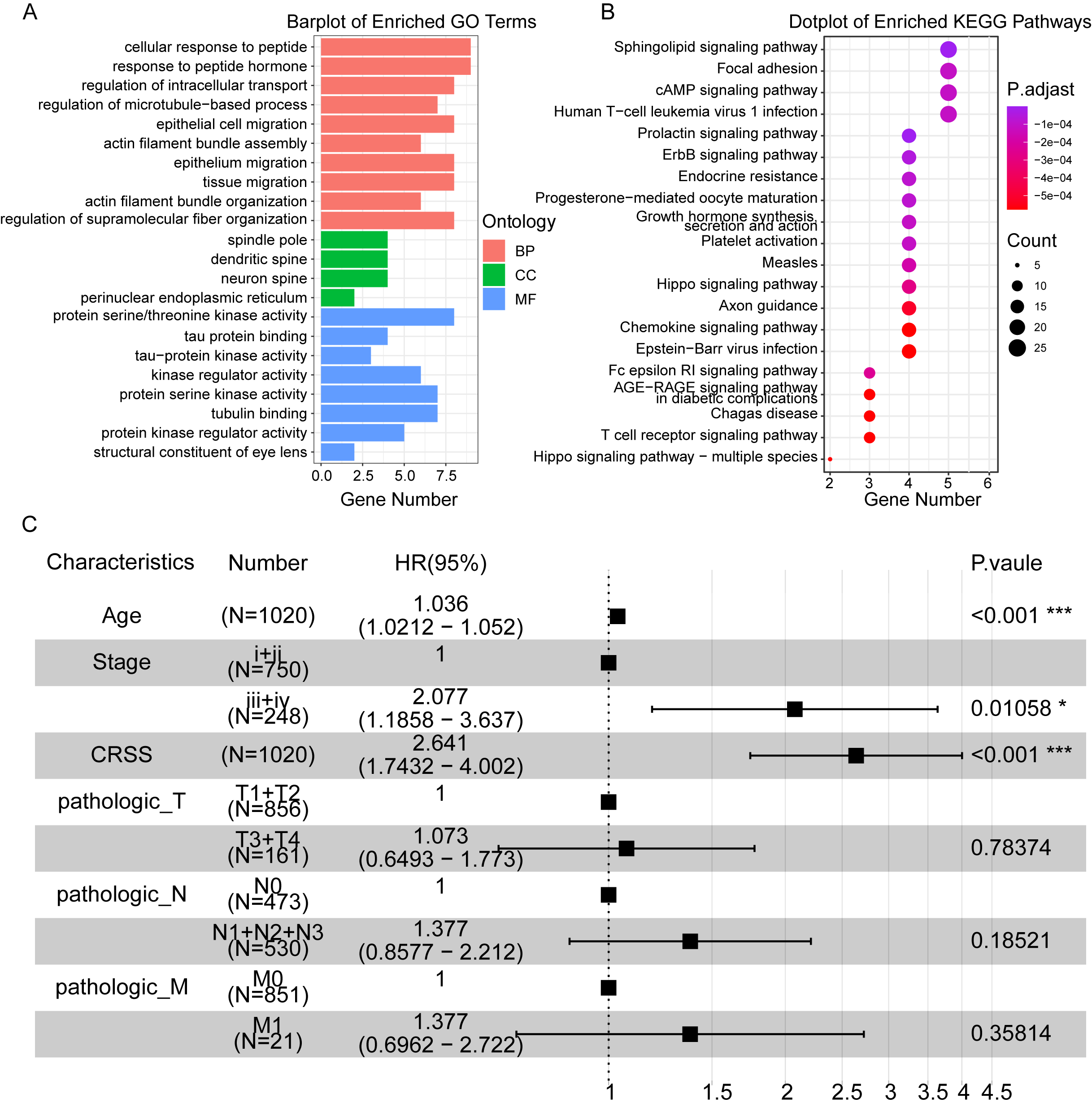

Supplement: Supplementary Figure 1 — Functional analysis of centrosome-related DEGs and multivariate Cox regression analysis of forest plots (A) GO enrichment of downregulated centrosome-related DEGs. (B) KEGG pathways of downregulated centrosome-related DEGs. (C) Multivariate Cox regression analysis of prognostic factors based on cross-validation and least partial likelihood deviation further argued for independent prognostic factors. [file Image_1.tif]

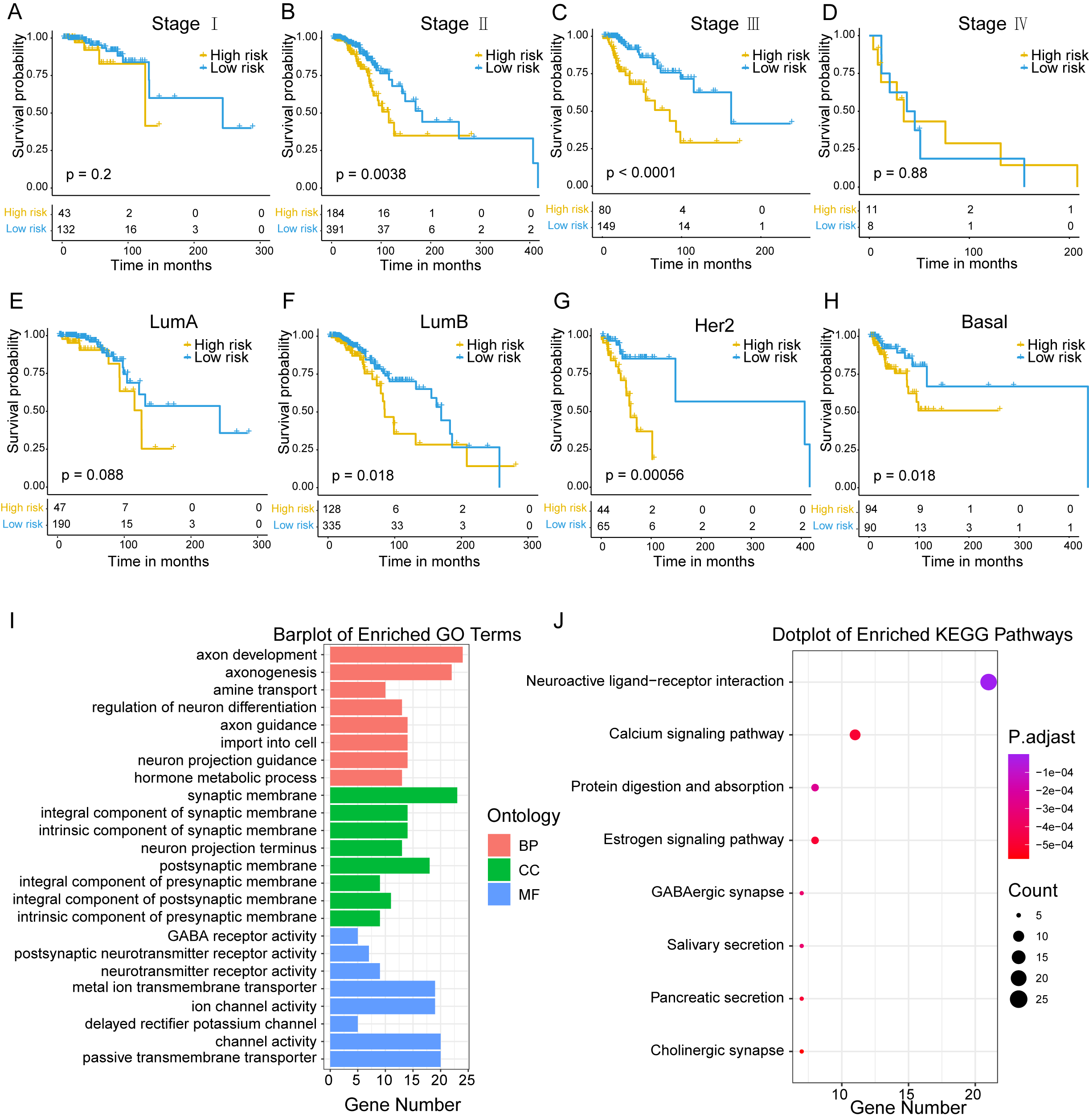

Supplement: Supplementary Figure 2 — Subgroup survival analysis and functional analysis between high/low-risk groups (A) Kaplan–Meier survival for OS curve of the patients in the high/low-risk groups in stage I breast cancer patients. (B) Kaplan–Meier survival for OS curve of the patients in the high/low-risk groups in stage II breast cancer patients. (C) Kaplan–Meier survival for OS curve of the patients in the high/low-risk groups in stage III breast cancer patients. (D) Kaplan–Meier survival for OS curve of the patients in the high/low-risk groups in stage IV breast cancer patients. (E) Kaplan–Meier survival for OS curve of the patients in the high/low-risk groups in LumA breast cancer patients. (F) Kaplan–Meier survival for OS curve of the patients in the high/low-risk groups in LumB breast cancer patients. (G) Kaplan–Meier survival for OS curve of the patients in the high/low-risk groups in Her2 breast cancer patients. (H) Kaplan–Meier survival for OS curve of the patients in the high/low-risk groups in Basal breast cancer patients. (I) GO enrichment of centrosome-related DEGs in high-risk subgroup. (J) KEGG pathways of centrosome-related DEGs in high-risk subgroup. [file Image_2.tif]

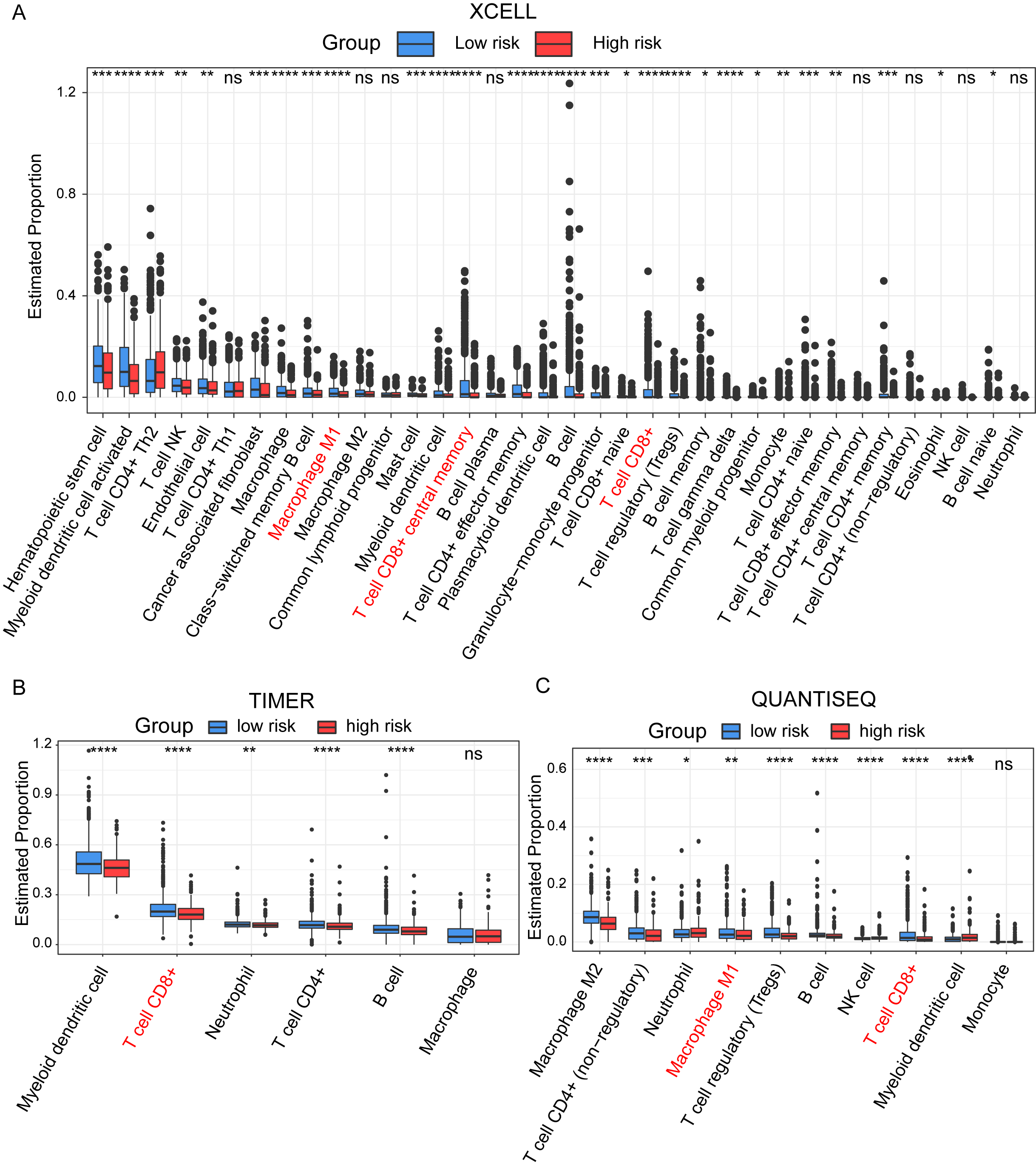

Supplement: Supplementary Figure 3 — Immune cell infiltration in high/low-risk subgroups. (A) Different estimated proportions of XCELL immune cell types in high/low-risk subgroups. (B) Different estimated proportions of TIMER immune cell types in high/low-risk subgroups. (C) Different estimated proportions of QUANTISEQ immune cell types in high/low-risk subgroups [file Image_3.tif]
